# Effects of Cadmium Exposure on Gut Villi in *Danio rerio*

**DOI:** 10.3390/ijms23041927

**Published:** 2022-02-09

**Authors:** Chiara Maria Motta, Emanuela Califano, Rosaria Scudiero, Bice Avallone, Chiara Fogliano, Salvatore De Bonis, Anja Raggio, Palma Simoniello

**Affiliations:** 1 Department of Biology, University Federico II, Via Cintia, 80126 Napoli, Italy; chiaramaria.motta@unina.it (C.M.M.); ema.califano@studenti.unina.it (E.C.); bice.avallone@unina.it (B.A.); chiara.fogliano@unina.it (C.F.); a.raggio@studenti.unina.it (A.R.); 2 Regional Agency for Environmental Protection of Latium (Arpa Lazio), Via Boncompagni, 00187 Roma, Italy; salvatore.debonis@arpalazio.gov.it; 3 Department of Sciences and Technologies, University of Naples Parthenope, Centro Direzionale, Isola C4, 80143 Napoli, Italy; palma.simoniello@uniparthenope.it

**Keywords:** cadmium toxicity, goblet cells, lectin staining, metallothionein expression, metallothionein localization, seric infiltration, villar degeneration, zebrafish

## Abstract

In aquatic organisms, cadmium exposure occurs from ovum to death and the route of absorption is particularly wide, being represented by skin, gills and gastrointestinal tract, through which contaminated water and/or preys are ingested. It is known that cadmium interferes with the gut; however, less information is available on cadmium effects on an important component of the gut, namely goblet cells, specialized in mucus synthesis. In the present work, we studied the effects of two sublethal cadmium concentrations on the gut mucosa of *Danio rerio*. Particular attention was paid to changes in the distribution of glycan residues, and in metallothionein expression in intestinal cells. The results show that cadmium interferes with gut mucosa and goblet cells features. The effects are dose- and site-dependent, the anterior gut being more markedly affected than the midgut. Cadmium modifies the presence and/or distribution of glycans in the brush border and cytoplasm of enterocytes and in the goblet cells’ cytoplasm and alters the metallothionein expression and localization. The results suggest a significant interference of cadmium with mucosal efficiency, representing a health risk for the organism in direct contact with contamination and indirectly for the trophic chain.

## 1. Introduction

Cadmium (Cd) is an extremely toxic heavy metal released in the environment due to a plethora of anthropic processes [1]. By inducing extensive cellular damage [2], it causes deleterious effects on animal behavior [3,4,5,6] and on adult and embryonic tissues [7,8], as well as altering gene expression [9,10,11]. The liver and kidney, the two main detoxifying organs, are particularly affected [12,13], but harmful effects have been reported on many targets, including the retina, brain, and muscles [14,15,16,17]. Cadmium is also an endocrine disruptor interfering with fecundity, oogenesis, and spermatogenesis [18,19,20,21].

Cadmium toxicity is higher in the aquatic organisms compared with land animals. In fact, in the former case exposure occurs from ovo to death and the route of absorption is particularly large, being represented by the skin and the gills. Both organs are markedly affected by the metal that causes severe morphological and molecular alterations [22,23]. A further route of absorption is the gastrointestinal tract through which contaminated water and/or contaminated preys are ingested, allowing metal distribution to the entire body via the bloodstream [24,25].

In the gastrointestinal tract, cadmium compromises the integrity of the microbiota, thus reducing the efficiency of this intestinal barrier and increasing the risk of inflammation, allergies, and metabolic disorders [26,27]. Cadmium has been reported to interfere with the gut mucosa. Degenerated nuclei, apoptosis, and altered lamina propria infiltrated with leukocytes have been reported in *Oreochromis niloticus* [28]; in *Ophiocephalus striatus*, intestine villi showed hydropic degeneration, swelling, and necrosis [29].

Cadmium also affects another important component of the gut: the goblet cells. Goblet cells are cells of the gastrointestinal surface epithelium specialized in the synthesis of mucus, a gel consisting of water, ions, and specific glycoproteins, termed mucins [30]. The mucus coating the gastrointestinal tract is considered the front line of the innate host defense, being a pivotal player of the immune system [31,32]. In the small intestine, mucus limits the number of bacteria that can reach the epithelium; in the large intestine, the inner mucus layer separates the commensal bacteria from the host epithelium [31]. The goblet cells also have a role in the presentation of oral antigens to the immune system [32]. The intestinal goblet cells secrete not only mucins but also several typical bioactive molecules [31]; the observation that mucus defects lead to diseases such as inflammatory bowel disease and or hyperglycemia underlines the importance of this barrier [33,34]. Changes in the expression of mucus genes and in mucin glycan structures occur in cancers of the intestine, contributing to diverse biologic properties involved in the development and progression of cancer [35,36]. Cadmium has been shown to damage the intestinal mucosal barrier making the body more susceptible to infection and inflammation [37,38,39,40]. This aspect is of particular concern in fish aquaculture, where the animals are fed with flour often derived from fish of little commercial value from particularly polluted areas [41]. This can greatly increase the occurrence of infections in farmed fish, resulting in increased use of drugs and antibiotics which, in turn, further reduce the quality of farmed fish.

In this paper, we focused on the effects of two sublethal cadmium concentrations on cells of the gastrointestinal surface epithelium with particular attention to changes in the distribution of glycan residues and the morphological features of the intestine. PAS and a panel of three lectins were used to highlight different sugar residues (WGA for d-N-acetyl-glucosamine; PNA for Galβ1-3GalNAcα1-Ser/Thr, galactose < N-acetyl galactosamine; RCA for galNAc, β-galactosamine). To evaluate the defense response of the mucosa, the expression of metallothionein (MT) was investigated. This is a multifunctional protein with cytoprotective properties against heavy metals and oxidative damage [42], also showing a cytoprotective role on the gastroduodenal mucosa [43]. The presence and localization of protein and MT mRNAs were determined by immunocytochemistry and in situ hybridization, respectively. The data demonstrate that cadmium modifies the presence and/or distribution of glycans in the brush border and cytoplasm of enterocytes and in the goblet cells cytoplasm. The results suggest a significant interference of cadmium, in a dose- and site-dependent manner, with mucosal efficiency. This effect could be a direct health risk for the organism exposed to the contamination and indirectly a risk for the trophic chain.

## 2. Results

### 2.1. Effects of Cadmium on Gut Morphology

**Figure 1:**
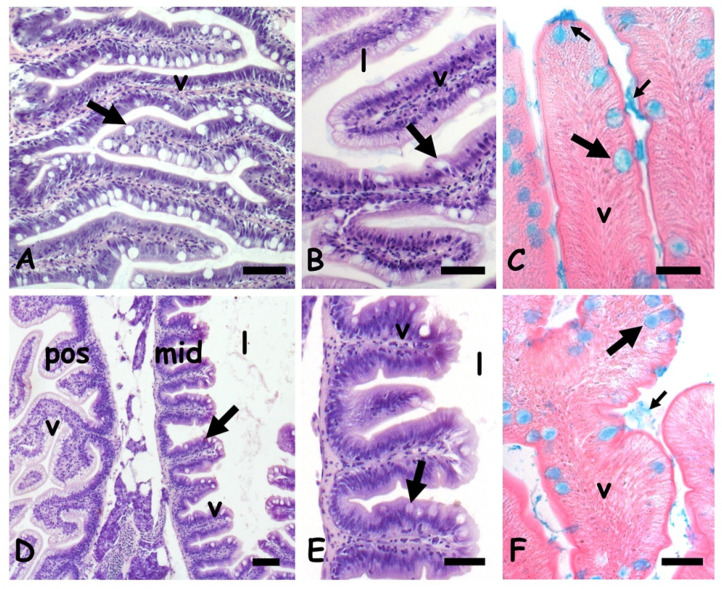
*Danio rerio* anterior (**A**–**C**) and mid (**D**–**F**) intestine. (**A**) Long villi (v) lined by a mucosal epithelium rich in goblet cells (arrow). (**B**) Detail of villi (v); lumen (l), goblet cell (arrow). (**C**) Goblet cells (large arrow) and luminal mucus (small arrow) positive to the Alcian blue staining. (**D**) Villi (v), goblet cells (arrow), and lumen (l) in posterior (pos) and mid (mid) gut. (**E**) Detail of midgut villi. (**F**) Goblet cells (large arrow) and luminal mucus (small arrow) positive to the Alcian blue staining. Hemalum-eosin staining (**A**,**B**,**D**,**E**); Alcian blue-eosin counterstaining (**C**,**F**). Bars: 100 µm (**A**,**B**,**D**), 50 µm (**C**,**E**,**F**).

In control zebrafish, the anterior intestine shows elongated villi covered by a columnar mucosa in which cylindrical enterocytes are interspersed with large and round goblets cells (Figure 1A,B). Their mucous content is clearly stained by Alcian blue (Figure 1C). In the mid intestine, the villi are broad and short and the lumen relatively large (Figure 1D,E). Mucosa goblet cells are numerous and clearly stained by Alcian blue (Figure 1F).

**Figure 2:**
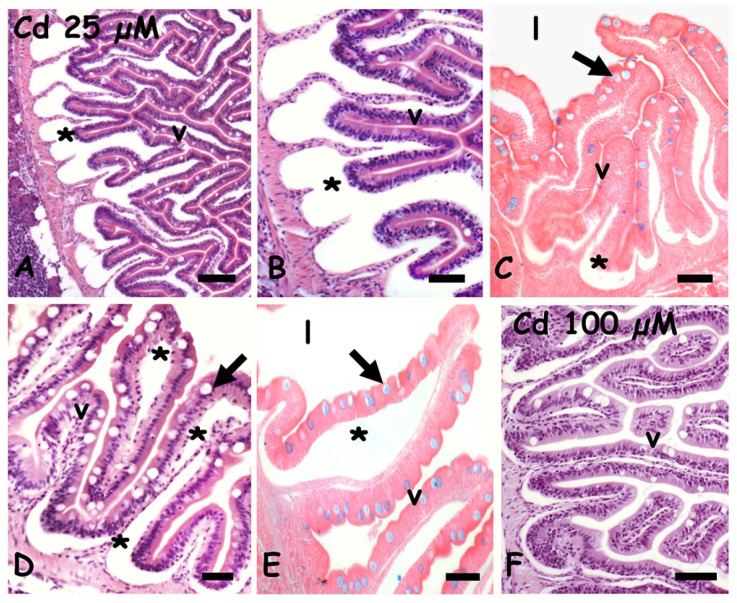
*Danio rerio* anterior (**A**,**C**,**F**) and mid (**D**,**E**) intestine exposed to waterborne cadmium. (**A**) Seric infiltrates (*) between connectives and mucosal epithelium. Villi (v). (**B**) Detail. (**C**) Poorly stained goblet cells (arrow) in villi (v) with seric infiltrates (*); gut lumen (l). (**D**) Seric infiltrates in lamina propria (*) and goblet cells (arrow). (**E**) Poorly stained goblet cells (arrow) in villi (v) showing extensive seric infiltration (*); gut lumen (l). (**F**) Intact villi (v). Hemalum-eosin staining (**A**,**B**,**D**,**F**); Alcian-blue-eosin counterstaining (**C**,**E**). Bars: 150 µm (**A**), 100 µm (**B**,**C**,**F**), 50 µm (**D**,**E**).

After exposure to 25 µM of cadmium, in both anterior (Figure 2A,B) and mid (Figure 2D) intestine, the mucosa is detached from the basal connectives by evident seric infiltrations extending in the lamina propria. The mucosal epithelium is apparently intact but goblets cells positivity to the Alcian blue is significantly reduced (Figure 2C,E). Exposure to 100 µM cadmium apparently has a far less toxic effect: the seric infiltrates are less relevant (Figure 2F) and the goblet cells are clearly stained by Alcian blue (data not shown).

### 2.2. Effects of Cadmium on Metallothionein Expression

**Figure 3:**
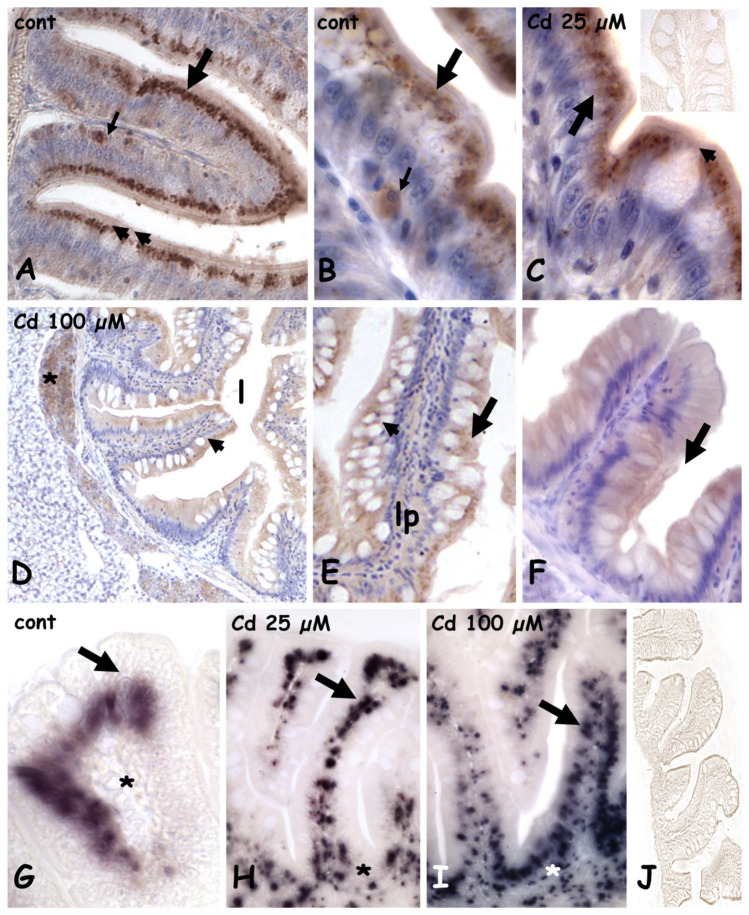
Metallothionein (**A**–**F**) and metallothionein mRNA (**G**–**J**) localization in the anterior and mid intestine in *Danio rerio* exposed to cadmium. (**A**–**C**) anterior gut; MT is in the apical cytoplasm of enterocytes (large arrow) and in occasional cells located close to the lamina propria (small arrow). Goblet cells are unstained (arrowheads). Anterior gut (**D**,**E**) and mid (**F**) gut; MT is uniformly dispersed in the cytoplasm of enterocytes (arrow). Goblet cells (arrowheads) are unstained. Lamina propria (lp), positive pancreatic cells (*). Inset in (**C**): negative control of immunocytochemistry. (**G**) anterior gut; MT mRNA is in enterocytes nuclei (arrow). Lamina propria is unstained (*). (**H**–**I**) anterior gut; dose-dependent increase in labeled submucosa and lamina propria nuclei (arrows). (**J**) Negative control of in situ hybridization. Hemalum counterstaining (**A**–**F**).

In controls (Figure 3A) and in animals exposed to 25 µM of cadmium, in both anterior (Figure 3B,C) and mid (Figure 3A) intestine, MT is abundant in the apical cytoplasm of the cylindrical enterocytes and in occasional sparse cells located close to the connectival lamina propria. Goblet cells and their content are always unstained (Figure 3A,C). After exposure to 100 µM of cadmium, in both anterior (Figure 3D) and mid (Figure 3E,F) gut, labeling is much less intense, and MT is uniformly distributed in the cytoplasm of enterocytes. No intensely labeled basal cells are observed, and goblet cells are always unstained (Figure 3E,F), similar to negative controls (Figure 3C inset).

MT messengers in the control gut are in the nuclei of enterocytes, while the lamina propria is negative (Figure 3G). After Cd exposure (Figure 3H,I), many small, labeled nuclei appear in the submucosa and in the lamina propria and their number is clearly dependent on metal dose. Negative controls are always unstained (Figure 3J).

### 2.3. Effects of Cadmium on the Carbohydrate Composition of Gut Cells

#### 2.3.1. N-Acetyl-Glucosamine Staining with Fluorescent WGA Lectin

**Figure 4:**
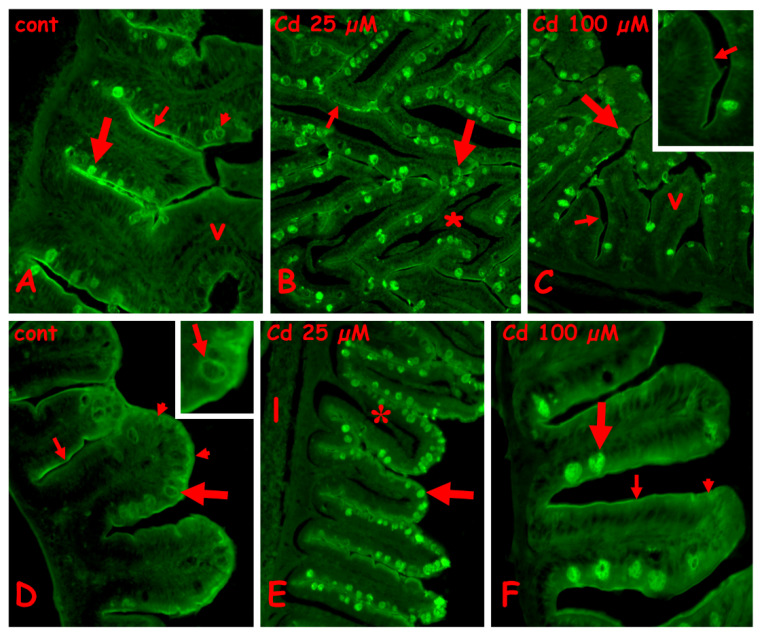
Effects of cadmium exposure on gut cells carbohydrate composition: staining for N-acetyl-glucosamine with fluorescent lectin WGA. Anterior (**A**–**C**) and mid (**D**–**F**) gut. (**A**) Villi (v) with labeled brush border (small arrow) and mucus vesicles in basolateral goblet cells (large arrow). In apical goblet cells labeling is cytoplasmic (arrowhead). (**B**) Intensely labeled apical goblet cells (large arrow) and brush border (small arrow). Seric infiltrate (*). (**C**) Labeled goblet cells in intact villi (v). Unstained brush border (small arrows). (**D**) Labeled brush border (small arrow), apical cytoplasm of enterocytes (arrowheads) and cytoplasm of goblet cells (large arrow and inset). (**E**) Labeled goblet cells (arrow) in villi with seric infiltrates (*). Unstained liver (l). (**F**) Labeled mucus vesicles in basal goblet cells (large arrow) and brush border (small arrow). Diffuse staining in the apical cytoplasm of enterocytes (arrowhead).

**Figure 5:**
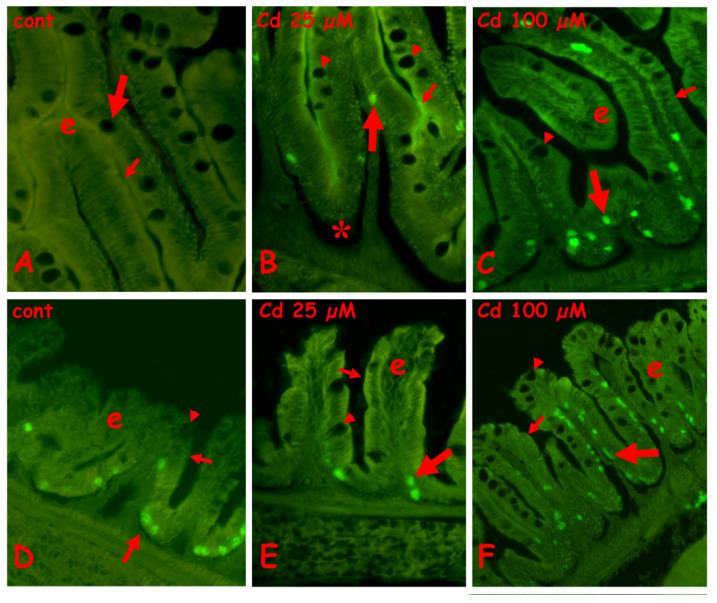
Effects of cadmium exposure on gut cells carbohydrate composition: staining for galactose with fluorescent lectin PNA. Anterior (**A**–**C**) and mid (**D**–**F**) gut. (**A**) Unlabeled enterocytes (e), goblet cells (large arrow), and brush border (small arrow). (**B**) Labeled brush border (small arrow) and scattered epithelial cells located at the base of the epithelium (large arrow). Note the seric infiltrate (*) and the unlabeled goblet cells (arrowheads). (**C**) Scattered labeled epithelial cells (large arrow) among unlabeled enterocytes (e), goblet cells (arrowhead) and brush border (small arrow). (**D**–**F**) Labeled cells (large arrows) in basal epithelium. Unlabeled enterocytes €, brush border (small arrow), and goblet cells (arrowhead). In (**F**), note the abundance of labeled cells.

In the control anterior gut, labeling is present on the brush border, the mucus of the goblet cells is located basally in the villi, and the cytoplasm of the goblet cells is located apically (Figure 4A). After exposure to 25 µM (Figure 4B) or 100 µM of cadmium (Figure 4C), in the anterior gut, all the goblet cells are labeled, on both cytoplasm and mucus vesicles. The brush border remains labeled after exposure to 25 µM of cadmium (Figure 4B), but after 100 µM of cadmium (Figure 4C), it shows long tracts completely negative to the lectin. The enterocytes are always unlabeled (Figure 5A–C).

In control midgut (Figure 4D), a diffuse staining is observed on the apical cytoplasm of enterocytes, on the brush border, and on the cytoplasm of the goblet cells (Figure 4D inset). After exposure to 25 µM of cadmium, all the goblet cells have labeled mucus granules (Figure 4E), while after exposure to 100 µM of cadmium, only the basal goblet cells are intensely labeled (Figure 4F). The enterocytes’ cytoplasm appears diffusely labeled and the brush border appears discontinuous (Figure 4E,F).

#### 2.3.2. Galactose Staining with Fluorescent PNA Lectin

In the control anterior gut, the villi are completely unlabeled (Figure 5A). After exposure to 25 µM of cadmium (Figure 5B), an intense labeling is present on the brush border and on scattered epithelial cells located close to the connectival lamina propria. The enterocytes show a moderate and diffuse apical positivity, while the goblet cells are completely unlabeled. A similar result is obtained in villi exposed to 100 µM of cadmium, in which, however, labeled cells are more numerous (Figure 5C).

In control midgut (Figure 5D), labeling is present on scattered epithelial cells located close to the lamina propria; enterocytes and goblet cells are unstained. After exposure to 25 µM (Figure 5E) or 100 µM of cadmium (Figure 5F), the labeled cells become more numerous, but no labeling appears on enterocytes, brush border, or goblet cells.

#### 2.3.3. Staining for N-Acetyl-Galactosamine with Fluorescent RCA Lectin

**Figure 6:**
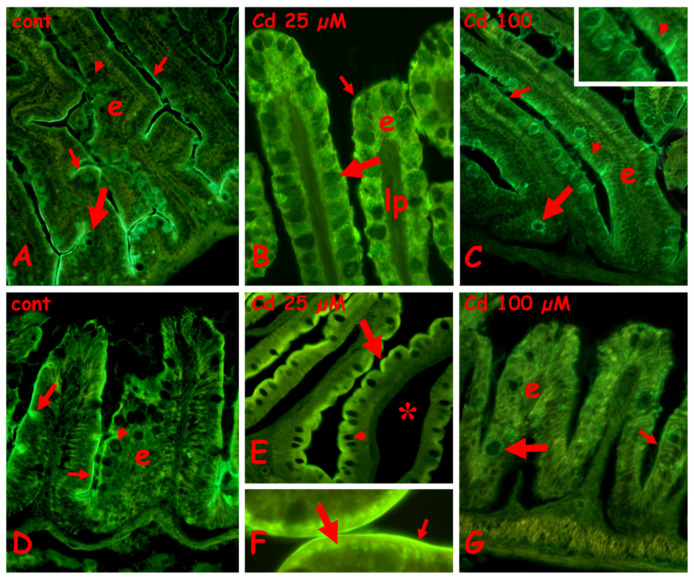
Effects of cadmium exposure on gut cells carbohydrate composition: staining for N-acetyl-galactosamine with fluorescent lectin RCA. Anterior (**A**–**C**) and mid (**D**–**F**) gut. (**A**) Villi with labeled brush border (small arrows) and cytoplasm of basal goblet cells (large arrow). The enterocytes (e) and the goblets cells located in the apical portion of villi (arrowhead) are unlabeled. (**B**) Diffuse labeling on enterocytes (e), brush border (small arrow), and goblet cells (large arrow). Unlabeled lamina propria (lp). (**C**) Labeled goblet cells cytoplasm (large arrow) and brush border (small arrow); enterocytes € show a moderate apical positivity (arrowhead and inset). (**D**) Labeled brush border (small arrow) and mucus vesicles in occasional goblet cells (large arrow); unlabeled enterocytes (e) and goblet cells (arrowhead). (**E**,**F**) Labeled apical cytoplasm of enterocytes (large arrow) and brush border (small arrow). Unlabeled goblet cells (arrowhead), seric infiltrate (*). (**F**) Detail of (**E**). (**G**) Unstained enterocytes (e), goblet cells (large arrow), and brush border (small arrow).

In control anterior gut, labeling is present on the brush border and on the cytoplasm of basal goblet cells. All the enterocytes and the goblet cells located along the villi are completely unlabeled (Figure 6A). After exposure to 25 µM of cadmium (Figure 6B), a diffuse staining is present on the enterocytes and, to a lesser extent, on mucus vesicles of goblet cells. In villi exposed to 100 µM cadmium, staining is on the cytoplasm of the enterocytes and goblet cells (Figure 6C and inset) and on the brush border.

In control midgut, labeling is present on the brush border and on the mucus vesicles of occasional goblet cells (Figure 6D). After exposure to 25 µM of cadmium, the apical cytoplasm of enterocytes appears intensely labeled (Figure 6E) due to the formation of a series of very fluorescent and regularly disposed dense bodies (Figure 6F). The brush border is also intensely labeled (Figure 6F). After exposure to 100 µM of cadmium, the villi are completely unlabeled (Figure 5G).

## 3. Discussion

In agreement with previous studies [28] cadmium exposure in *Danio* resulted in a disruption of villar organization. In particular, the mucosal epithelium detaches from the underling basal mucosa due to a massive edema [44]. A possible cause is the interference of cadmium with ion and nonelectrolytes transport and/or with adhesion proteins [45]. No degeneration or swelling or loss of structural integrity is observed at the tip of the villi [29].

As expected, the damage is more extended at the lower concentration; the same evidence was obtained in the retina and kidney tubules [13,15]. Higher toxicity at a lower concentration can be explained considering that cadmium is a calcium and zinc mimetic and, by entering the cell at a low concentration, it may remain invisible to the detoxifying mechanisms [46,47]. Indeed, in villi of 25 µM Cd-treated animals, metallothioneins remain concentrated in the enterocyte’s cytoplasm as in control villi, while MTs reduce significantly after 100 µM Cd exposure, presumably since it was used to detoxify the mucosa.

Another possibility explaining the reduced cytosolic MTs after 100 µM Cd is that the proteins were released into extracellular compartments to mediate the inflammatory response [48]. In effects, in baso-lateral submucosa and in the lamina propria, Cd exposure causes the appearance of several small and scattered cells that, for position and size, would represent elements of the gut-associated lymphoid tissues (GALT) [49]. Infiltration with inflammatory cells has been reported in submucosa of several teleosts after toxic insult [50] and GALT cells, lymphocytes, plasma cells, macrophages, and granulocytes are all known to produce MTs [51,52,53]. The infiltration with GALT cells is also supported by the evidence obtained by staining with PNA lectin, specific for galactose. As reported in mice and fish [54,55], lymphocytes expose galactosyl sites that represent a signal for mitogenic activation.

As far as goblet cells are concerned, Cd at both concentrations does not determine a massive release of mucus in the gut lumen. Mucins, however, show a slightly reduced affinity for Alcian blue, suggestive of a change in composition [56]. Investigation with the panel of lectins indicates an increase in N-acetyl-glucosamine, in both anterior and midgut, but no significant changes in galactose or N-acetyl-galactosamine. This latter sugar residue, however, moderately decreases in goblet cells cytoplasm, in mid but not anterior gut. The three lectins do not give an exhaustive overview of what happens to glycosylation in the gut mucosa after waterborne cadmium administration but clearly indicate that significant changes occur. A more complete panel of lectins will contribute to clarifying in more detail the target(s) of Cd toxicity. In effect, interference of cadmium on the glucidic pattern has already been demonstrated in several tissues, including muscles [57], oocytes [21], and the liver [12]. Changes have been related to the significant variations induced by the metal in gene expression [9,10,11]. In the future, a chemical/structural study of the isolated mucins could provide more information.

Mucus release by goblet cells is finely regulated and of fundamental importance for gut functionality [31]. Its activity is assisted by the enterocytes’ brush border, a structure rich in glycoconjugates and in NAc-galactosamine, NAc-glucosamine, mannose, glucose, and sialic acid in particular [58]. Cadmium administration seems to modify sugar composition: a brush border positive in controls becomes discontinuously positive for N-acetyl-glucosamine distribution. It does not contain galactose, but becomes very rich in N-acetyl-galactosamine at a lower Cd dose and completely depleted at a higher dose.

The enterocytes’ cytoplasm behaves differently from the brush border. It is and remains negative for N-acetyl-glucosamine and galactose [59], even after Cd administration. In contrast, it appears moderately richer in N-acetyl-galactosamine, but only at the lower Cd dose, 25 µM, and with a different distribution in the anterior and midgut. In the former, the sugar is dispersed in the entire cytoplasm, while in the latter, it is concentrated in the apical cytoplasm and is apparently organized in vesicles. No explanation could be found since N-Ac-gal in fish is reported scarcely present and located mainly in goblet cells [58].

Information about the distribution and function of the different sugar components in the gut is still under investigation [59]. Glycoconjugates participate in food absorption and in the defense against parasites and toxins [58]; mucins control water absorption and lubrication. Any change in composition will significantly affect gut efficiency, growth, and eventually animal survival. It is not the case that glycoconjugates differ in the different tracts of the intestine and in different species according to the diet [60].

## 4. Material and Methods

### 4.1. Animals and Cadmium Treatments

Adult and healthy male zebrafish (average wet weight 1.50 ± 0.22 g) were obtained from a specialized local supplier and maintained under standard conditions in 50 L tanks. After a two-week period of acclimation, 24 animals were randomly allotted to three 5 L experimental tanks.

The first eight animals were left untreated (control), the second eight were exposed to 25 µM CdCl_2_, and the third group of eight animals to 100 µM CdCl_2_. Cadmium dosage was chosen so to mimic the exposure occurring in a typical polluted water basin [57]. Treatments lasted 96 h, under static condition: no further CdCl_2_ was added during the experimental period so to avoid overlapping the effects due to daily renewing [21]. No sign of sufferance or mortality were recorded during treatment, confirming previous evidence [15].

### 4.2. Tissue Sampling, Processing, and Staining

The animals were sacrificed by an overdose of ethyl 3-aminobenzoate methanesulfonate (MS-222, Sigma Aldrich, Milano, Italy) solution (300 mg/L). Samples of gut tissues were dissected and immediately processed for light microscopy. In brief, tissues were fixed in Bouin’s solution (4 h), dehydrated in ethanol, and embedded in paraffin wax. Sections (6 μm) were mounted on glass slides and stained with hematoxylin-eosin, to show general morphology, or with Alcian blue (1%, pH 2.5), to localize carboxylated and/or sulfated type of acidic mucins in goblet cells, brush border, and gut lumen [61].

Sections of the anterior intestine and of the anterior mid intestine [62] were selected and stained with a panel of three lectins conjugated with FITC (Vector Laboratories Inc.; Burlingame, CA, USA; 2 mg/mL) to reveal the presence of specific carbohydrate residues. Lectin WGA (*Triticum vulgaris*, wheat germ) was used for d-N-acetyl-glucosamine (glcNAc); PNA (*Arachis hypogea*, peanut agglutinin) for Galβ1-3GalNAcα1-Ser/Thr; and galactose < N-acetyl galactosamine (Gal) and RCA (*Ricinus communis*) for galNAc and β-gal (galNAc).

Slides were rinsed in PBS (0.2 M, pH 7.2–7.4) for few minutes and incubated with lectins at a concentration of 10 µg/µL in PBS for 15 min at room temperature in a moist chamber in the dark. After rinsing in PBS, binding sites were visualized under a UV light (excitation maximum at 495 nm and emission maximum at 515 nm). Labeling was defined as positive or negative by the same observer [63]. Negative controls were prepared by incubating slides with the lectins and the specific competing sugar or by omitting the lectin in the reaction; no autofluorescence was observed.

### 4.3. Metallothionein Localization

Immunohistochemical reactions were performed on deparaffinized gut sections as previously described [64]. To unmask the antigens, slides were microwaved at 750 W for 15 min in citrate buffer (10 mM, pH 6) and washed in H_2_O_2_ to reduce peroxidase non-specific bindings. Sections were exposed to the primary MT antibody (rabbit polyclonal antibody, Santa Cruz Biotechnologies, Inc.; Santa Cruz, CA, USA), diluted 1:200 in PBS/BSA/Tween-20 0.5% buffer, overnight at 4 °C. Then, they were repeatedly washed in PBS and binding sites were revealed with a secondary peroxidase-conjugated antibody (1:400 in PBS, Sigma Aldrich, Milano, Italy) for 90 min at room temperature followed by a tertiary anti-PAP antibody (1:100 in PBS) and developed with DAB tablets. Negative controls were prepared by omitting the primary antibody or by carrying out the reaction on sections digested with protease (data not shown). The sections were counterstained with hematoxylin, dehydrated, cleared in xylene, and mounted with a coverslip. Images of sections were acquired using a Zeiss Axioskop microscope equipped with a camera.

For in situ hybridization, sections (5–7 µm) of gut samples were placed on superfrost glass slides (Menzel-Glaser, Bad Wildungen, Germany), fixed in paraformaldehyde 4% in PBS (137 mM NaCl, 2.7 mM KCl, 10 mM Na_2_HPO_4_, 2 mM KH_2_PO_4_) pH 7.4 for 20 min, and incubated in PK buffer (Tris-HCl, 0.2 M, pH 7.4, EDTA 0.01 M, pH 8, proteinase K, 10 µg/mL, H_2_O_depc_) at 37 °C for 15 min. After washing in PBS, they were incubated at 42 °C for 90 min in a pre-hybridization mix containing formamide, SSC 4× and 1× Denhart’s solution. Hybridization was carried out at 42 °C overnight using the dig-labeled cDNA probe encoding a piscine MT fragment [65]. Sections were washed in SSC 2×, in Buffer I (Tris-HCl 0.1 M, pH 7.5, NaCl 0.1 M, H_2_O_depc_) and in Buffer I containing blocking reagent (0.5%). Digoxigenin was revealed by incubating sections overnight with an AP-conjugated anti-dig antibody diluted 1:400. Slides were washed in Buffer I, incubated with levamisole-Tween20 1× for 15 min and exposed with BM-Purple. Dig-labeled MT cDNA probe was generated by PCR using the DIG High Prime DNA labeling and detection starter kit I (Roche). For negative control, the hybridization solution did not contain the cDNA probe.

## 5. Conclusions

In summary, this study demonstrated that cadmium affects the gut on different levels. First, histological analysis shows cadmium-induced villar degeneration and seric infiltration. Exposure to cadmium, regardless of the dose, significantly interferes with the presence and/or distribution of glucids in the different cell types at different levels of the gut. Conversely, changes in MT expression and localization are dose-dependent, being detectable only after exposure to the higher dose. Although the relationship between changes in mucus lectins and the well-known changes in the intestinal microbiota induced by cadmium remains speculative, it is conceivable that the glucidic components of mucins play a key role in the toxic mechanism; hence, future studies on the chemical properties of mucins will help us to understand it. Taken together, these findings highlight the health risk of environmental and dietary exposure to cadmium for animals and people living in highly polluted habitats.

## Data Availability

The authors confirm that the data supporting the findings of this study are available within the article.
